# ISO26262-Compliant Inductive Long-Stroke Linear-Position Sensors as an Alternative to Hall-Based Sensors for Automotive Applications

**DOI:** 10.3390/s23010245

**Published:** 2022-12-26

**Authors:** Hyung-Sok Lim, Choul-Young Kim

**Affiliations:** Department of Electronics Engineering, Chungnam National University, Daejeon 34134, Republic of Korea

**Keywords:** airgap effect, automotive position sensor, external magnetic field, Hall position sensor, gradiometric 3D Hall sensor, inductive position sensor, ISO26262, long-stroke position, robust magnetic immunity, target loss

## Abstract

To ensure safety, vehicle companies require position sensors that maintain accuracy and avoid target loss even in harsh automotive environments. Most vehicle position sensors are Hall-based, but even improved gradiometric 3D Hall sensors using the arctangent operation are vulnerable to external magnetic fields (EXMFs) and encounter difficulty at long-stroke (LS) positions. An ISO26262-compliant inductive position sensor (IPS) employing a 3.5 MHz-induced magnetic field source (much higher in frequency than vehicle–environment EXMFs) is proposed in this study as an alternative. To meet the safety goal, a threshold LS distance of 12 mm was set. Then the IPS was compared to existing Hall-based sensors. The B field of the existing 3D sensor was weak at LS and the airgap between sensor face and magnet target caused a large error in accuracy, whereas the IPS was not affected by LS. Because of its high excitation frequency, the IPS was also largely unaffected by EXMFs, as was demonstrated by ISO11452-8 and 0.1 T immunity tests. The proposed IPS outperformed existing 3D Hall sensors, achieving stable accuracy within ±0.85% for different airgaps (1.5–2.5 mm) and proving robust to magnetic and LS effects.

## 1. Introduction

Inductance, capacitance, and magnetic Hall effect measurements were all used in position sensor designs [1,2,3,4,5,6,7,8]. In automobile systems, most position sensors are Hall-based. As vehicle systems are increasingly automated [9], such as an electric vehicles (EVs), battery electric vehicles (BEVs), hybrid electric vehicles (HEVs), plug-in hybrid electric vehicles (PHEVs), and fuel cell electric vehicles (FCEVs) [10], become more commonplace, the sensitivity of vehicle position sensors to the external magnetic field caused by the high currents in the harness surrounding engine, motor, and battery increasingly affected safety and accuracy, especially near the long-stroke (LS) position. In particular, the tight safety specifications in ISO26262 [11], as well as other industry requirements, become difficult to meet. The accuracy of a 12 mm LS position sensor is generally required by international manufacturers to be within ±1% at full-scale output (FSO) [12]. Because fail-safe operational systems use redundant system functions in an architecture that allows transition to a backup system if a malfunction occurs in the primary system, global IC component suppliers are beginning to offer double and triple dies within a single package to support the need for redundancy without occupying more physical space [13,14]. Additionally, they try to offer a safety function IC for the external magnetic field [7,12,15,16].

Globally, 80% of position sensors are Hall-based. Many algorithms were proposed to improve Hall position sensors, including the chopper-stabilized differential switched-capacitor-filtering technique [17], CMOS chopper amplifier [18], 2D CMOS integrated Hall sensor [19], circular vertical Hall (CVH) technology [6,20,21], and three-dimensional (3D) magnetic sensors [22,23,24,25]. Of these, the gradiometric 3D Hall sensor and CVH Hall-based sensor are the newest. Both are commonly employed as long-stroke position sensors worldwide and will be used in this study as the standards of comparison.

Even the gradiometric 3D Hall-based sensors MLX90333, A31315, and A1335 [24,25,26] exhibit failure modes in external magnetic fields (EXMFs) and LS application. As Hall-based sensors use permanent magnets and react to magnetic fields with frequencies under 1 MHz, giant magnetoresistance (GMR) is considered more sensitive [27,28,29]. In the case of the 3D GMR position sensor, although the performance is good in terms of accuracy, it results in much worse results in terms of stability and safety. In the case of the recent 3D Hall sensor, the safety point of view, even redundant two-die or triple-die integrated Hall-based sensors using the highly accurate arctangent algorithm [30], can lose main target data in EXMFs or with an LS over 12 mm. Therefore, even double- or triple-die integrated designs are eventually useless because the target is lost, violating the main safety goal.

This study’s main novelty is the development of an over-12 mm LS sensor that is accurate and does not miss the target position. Most previous designers tried to make long-position sensors based on the Hall effect, but such sensors cannot meet the accuracy and safety requirements for harsh automotive environments with EXMFs. Therefore, this paper focuses on comparing 3D Hall sensors to an IPS optimized for long-stroke position sensing and safety in automotive–environment magnetic fields.

Setting a threshold distance of 12 mm for the LS position, we compared current 3D Hall sensors, including ones using arctangent methods, to another type of sensor, the inductive position sensor (IPS) [31]. We propose the following hypotheses:

First, we hypothesize that an IPS is much more robust to external magnetic fields when a high frequency (e.g., 3.5 MHz) is used with the induced raw magnetic field. Whereas EXMFs typically have frequencies under 150 kHz, the induced raw magnetic field can have a frequency in the range of 2.2–5.6 MHz. This range is not used under automotive electromagnetic compatibility (EMC) specifications, which apply under 2 MHz and over 5.9 MHz, according to the CISPR 25, 3rd edition frequency range (e.g., Hyundai Kia Motors Company EMC standard ES-96200) [9]. The frequency we choose is 3.5 MHz. Because this is in the middle of the unused EMC frequency band, the IPS does not lose the target position when exposed to an external magnetic field (under 150 kHz); at the same time, automotive EMC requirements can also be satisfied. This is the most important factor for automotive safety and ISO26262 compliance.

We also hypothesize that, in terms of stroke position sensing accuracy, the IPS error is due entirely to the airgap (AG) between the target and the printed circuit board (PCB) pattern. Therefore, it should be more accurate than a 3D Hall sensor, which has an error related to exponential decay of the waveform by target distance [32], as well as the AG change between sensor face and target.

To test these hypotheses, we compared the accuracy of IPS and Hall-based sensors with a 12 mm LS condition and AGs ranging from 1.5 to 2.5 mm. To quantify the effects of EXMFs, we performed ISO11452-8 standard tests with an arctangent 3D Hall sensor (MLX90333) [24], arctangent 3D IPS (ZMDI 5201) [31], and arctangent CVH 3D (A1335) sensor [26]. Furthermore, the performances of the arctangent IPS and 3D arctangent Hall sensor were compared under a stronger external magnetic field. The accuracy of both sensors was analyzed for different AG widths (sensor face to target face) and stroke moving lengths ranging from 0 to 12 mm.

## 2. IPS Theory

In comparing the IPS and 3D Hall sensors, we begin by assuming that, even if calibration and optimization are performed with a 3D Hall sensor, a 3D Hall sensor’s accuracy result would have a 5% or more not good performance at the long-stroke distance (12 mm).

**Figure 1:**
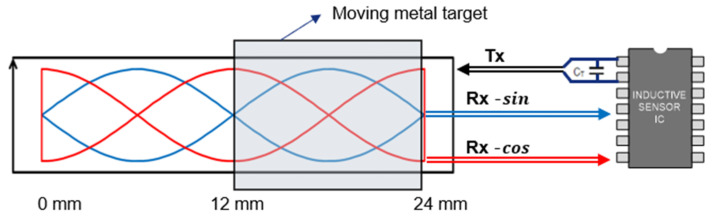
3.5 MHz tuned excitation by integrated oscillator with pin capacitor $C_{T}$.

According to Faraday’s law,

(1) $$Emf=–N_{rx}\;\frac{d\Phi_{B}}{dt}=–N_{rx}\frac{d\left(BAcos\theta \right)}{dt},$$

where $N_{rx}$ is the number of turns in the two receiving (*Rx*) coils (which have a 90° phase difference and may be considered sine and cosine loops) and $\Phi_{B}$ is the magnetic flux coupling to the coil. Equation (1) calculates the electromotive force (*Emf*) of the area enclosed by the transmitting (*Tx*) loop, as shown in Figure 1; the *Tx* pattern is a PCB pattern enclosing the receiving sine and cosine patterns. Using Maxwell’s law of induction,

(2) $$-\frac{\partial }{\partial t}\Phi_{B}=\frac{d}{dt}\oint_{C}\vec{E}\cdot d\vec{l}=-\frac{d}{dt}\oint_{C}\vec{B}\cdot d\vec{A}\;\;=-N_{rx}\;\frac{dB}{dt},\;$$

where $\vec{E}$ is the electric field along a closed loop induced by the changing $\Phi_{B}$ in the region encircled by the *Tx* loop.

Faraday’s law can be combined with Ampere’s law, as follows:

(3) $$B=\frac{\mu_{0}N_{Tx}I_{Tx}}{l},$$

where l is the *Tx* pattern length, $\mu_{0}$ is the permeability of AG (assumed to be that of free space), $N_{Tx}$ is the number of turns in the *Tx* pattern, and $I_{Tx}$ is the current in the *Tx* loop.

**Figure 2:**
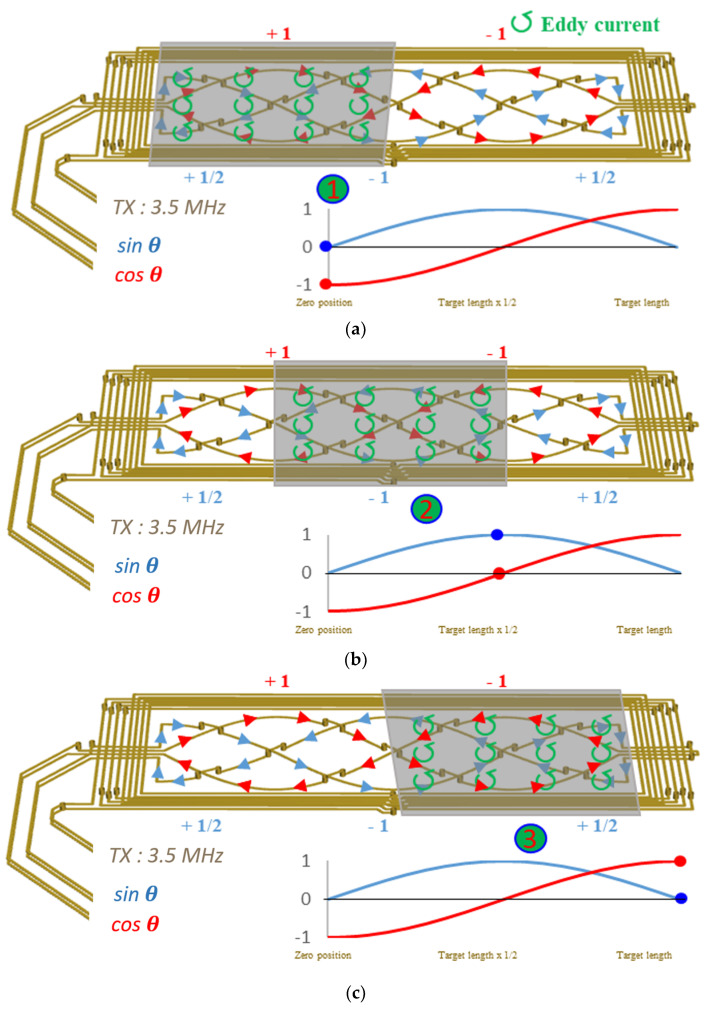
Inductive position sensor showing a 3.5 MHz high-frequency transmitting (*Tx*) loop and receiving (*Rx*) coils (*sin* θ and *cos* θ), induced current flow, and eddy currents: (**a**) zero position (0 mm) of the moveable target; (**b**) half-way position (6 mm); (**c**) final position (12 mm).

Substituting (3) into (2), with (4) we obtain (5)

(4) $$V_{tx}=i_{tx}\left(t\right)\;\omega \;L_{tx},\;\;\left(\omega =2\pi f,\;f=3.5\;\mathrm{MHz}\right)$$

where $V_{tx}$ is the exciting AC voltage of the primary coil. Then, at

(5) $$\Psi_{\Phi \_rx\_t}=-N_{tx}N_{rx}\;\frac{\mu_{0}}{l}dA_{target}\;\frac{di{\;}_{tx}}{dt}\;2\pi f\;L_{tx},$$

where $\Psi_{\Phi \_rx\_t}$ is the induced EMF from the *Tx* coil with parameters $N_{tx},\;\frac{di_{tx}}{dt}$, and $L_{tx},$ and the frequency of the *Rx* patterns (sine or cosine) is 3.5 MHz. If Urx1(θ) and Urx2(θ) are the sine- and cosine-pattern *Rx* voltages induced by the *Tx* coil,

(6) $$Urx1\left(\theta \right)=Pv_{a}\;sin\;\theta ,\;Pv_{a}=Pv\left(\;\Psi_{\Phi \_rx\_t}\right)+Pv\left(\tilde{\Psi_{\Phi \_rx\_t}}\right),\;$$

(7) $$Urx2\left(\theta \right)=Pv_{a}\;cos\;\theta ,\;Pv_{a}=Pv\left(\;\Psi_{\Phi \_rx\_t}\right)+Pv\left(\tilde{\Psi_{\Phi \_rx\_t}}\right),\;$$

where $Pv_{a}$ is the total radiometric power at the actual position as in Figure 2a–c. $Pv_{a}$ represents the total power induced by the *Tx* coil, except for $Pv\left(\tilde{\Psi_{\Phi \_rx\_t}}\right)$, the induced power of the moving metal target. The 1 and –1 positions on the *y*-axis of Figure 2a–c represent $Pv_{a}$ when $Pv\left(\tilde{\Psi_{\Phi \_rx\_t}}\right)$ of the metal target is zero by the skin effect, which is governed by

(8) $$\delta =\sqrt{\frac{1}{\pi f\sigma \mu }}\;,$$

where δ is the skin depth of the target, f is the oscillating frequency (3.5 MHz), σ is the conductivity of the moving target, and μ is the magnetic permeability.

Finally, the linear position is calculated using arctangent function, in the same way as in the arctangent 3D Hall sensor:

(9) $$atan\left[\frac{Pv_{a}\;sin\;\theta }{Pv_{a}\;cos\;\theta }\right]=atan(tan\theta )=\theta ,$$

(10) $$\theta =atan\;\left(\frac{Urx1\;\theta }{Urx2\;\theta }\right).$$

**Figure 3:**
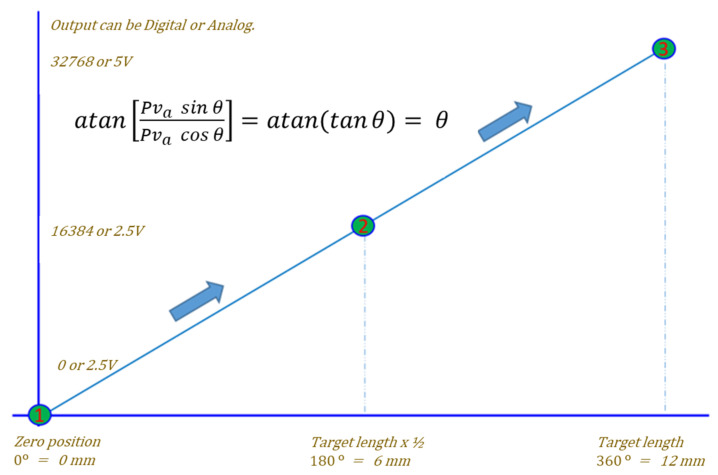
Arctangent computing with linearization result of Figure 2.

The resultant angle θ can be interpreted as the ratio between the actual position and the target length, multiplied by 360°, according to the CORDIC trigonometric computing technique [33] (Figure 3). After linearization, the results can be transferred as analog or digital output (SENT, I2C, PWM).

Using the arctangent method, the IPS performs a calibration process that includes a slope and offset linearity compensation with zero angle compensation. Through this process, the IPS can cover not only mechanical, but also any manufacturing errors [34].

To reduce the effect of external magnetic fields on Hall effect-based position sensors, it is necessary to avoid overlap with their frequencies. The frequency band of external magnetic fields in vehicles, as per the automotive EMC test standard (ISO11452-8), is under 150 KHz, but it is impossible.

We used a ZMDI 5201 IC as a representative IPS. This IC contains an integrated LC oscillator and external Tx pin capacitor $C_{T}$ that can be tuned to an excitation frequency in the 2.2–5.6 MHz range (Figure 1). We chose 3.5 MHz, a center frequency that can fulfill the requirements of a vehicle EMC test of radiated emission (RE) and conducted emission (CE), as defined by CISPR [9]. To set IPS consisting of an LC tank receiver, an LC filter with a high Q factor [35] (over 10) was employed for an operating frequency of 3.5 MHz. Eventually, IPS operates only by a 3.5 MHz B field induced in the PCB pattern. This means the accuracies of the long-stroke position sensors proposed here are not influenced by EXMFs under 150 kHz (Out of 3.5 MHz frequency range). Therefore, an IPS that can meet the ISO26262 position sensor safety goal should not miss the target position in an automotive environment.

**Figure 4:**
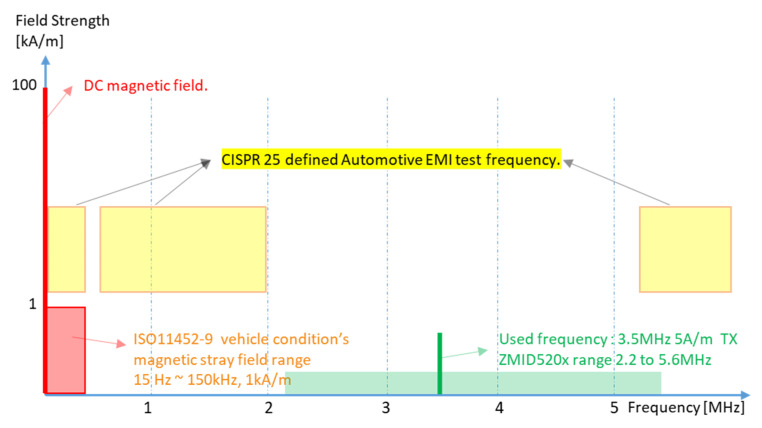
Proposed inductive position sensor excitation frequency of 3.5 MHz (BOLD green pk) compared to the other frequency ranges. CISPR automotive test frequency (yellow), vehicle condition’s external magnetic field range (red), and ZMID 520× IPS sensor’s possible tuning frequency range (green).

In other words, because 3.5 MHz lies in a completely different, higher frequency range than the vehicle’s harsh magnetic frequency range (which is under 150 kHz), using it completely avoids interaction with the extra magnetic field (Figure 4).

Another key factor in the performance of an LS position sensor is accuracy. The effects of AG and stroke length must be reduced to improve the accuracy of the Hall effect-based position sensor. To address this problem, the β factor (which exhibits an exponential decrease with an increase in the airgap [32]) must be considered:

(11) $$\;V_{H}=\frac{K_{H}\beta I}{z}.\;$$

Here, *V_H_* is the Hall voltage, *K_H_* is the Hall constant or Hall effect co-efficient, *β* is the magnetic flux density (perpendicular to flux), *I* is the current flowing through the conductor, and *Z* is the thickness of the conductor.

To reduce the error, both the IPS and 3D Hall sensors adopt the arctangent function θ of Equations (9) and (10). A benefit of this is constancy of the phase for different airgaps [30].

**Figure 5:**
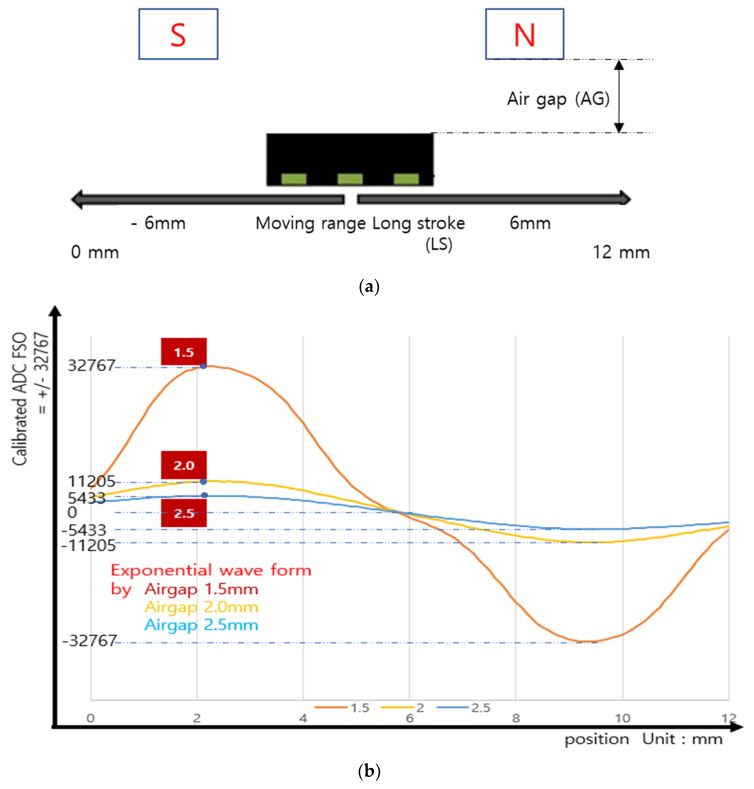
(**a**) Illustration of the 3D Hall sensor airgap and moving range. (**b**) 3D Hall sensor raw data for change in analog–digital converter (ADC) amplitude with ±6 mm moving range and various airgaps.

However, the *β* in Equation (11) for a 3D Hall-based sensor’s raw mapping data have an exponentially declined wave form by the airgap and target distance [32]; at the LS position of 12 mm, the raw data show a dramatic reduction, almost to noise level. Figure 5a illustrates the definitions of a 3D Hall sensor’s moving range and airgap. Figure 5b shows raw data for the change in airgap alone. With an airgap of 2.5 mm, the analog-to-digital converter (ADC) amplitude is reduced by almost 83% from its value with a 1.5 mm airgap. Choosing the maximum airgap, the arctangent 3D Hall sensor’s raw output at the left side (–6 mm), right side (+6 mm), or center position is almost the same as the noise level; these distances are the farthest points from the Hall sensor face.

**Figure 6:**
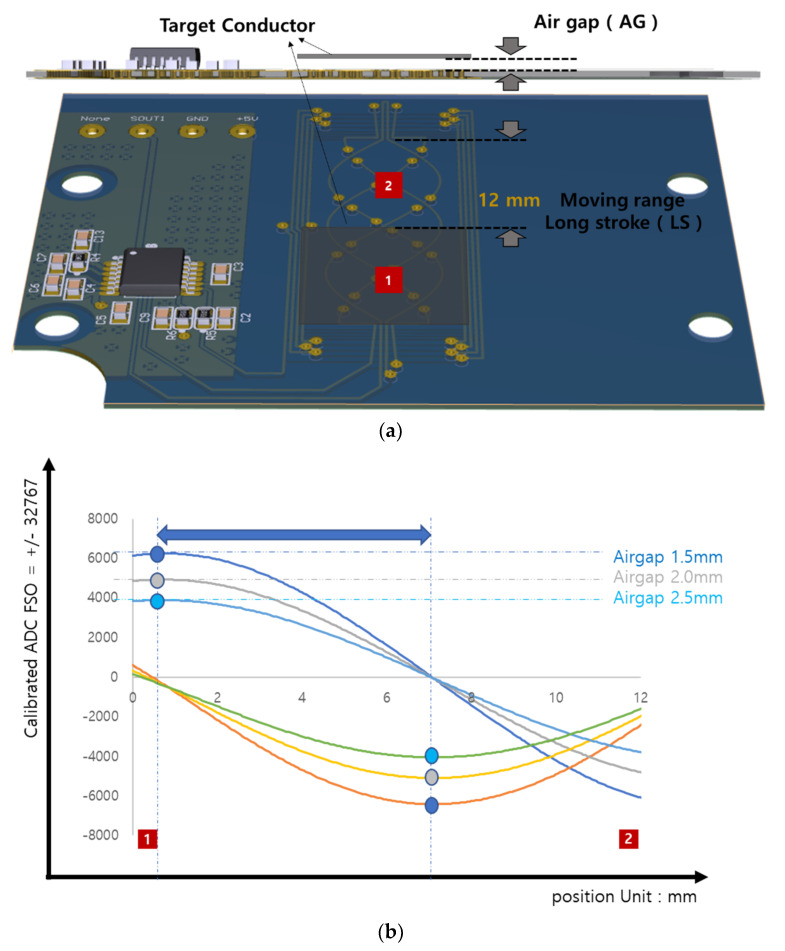
(**a**) Inductive position sensor (IPS)-target moving range (12 mm) on the PCB inductive position. (**b**) IPS raw data for change in analog–digital converter (ADC) amplitude for position range 0–12 mm and various airgaps (1.5, 2, or 2.5 mm), raw phase value of sinθ or cosθ, and the blue arrow is always the same by airgap change.

In the IPS case, the raw output fell from 6000 to 4000 with the increase in airgap, only a 6% ((6000–4000)/32,767) change (Figure 6b). Amplitude of the raw data did not change significantly with stroke position, whether at 0 mm or at the 12 mm target position (Figure 6a). Compared to the 83% raw data change with the Hall sensor, the 6% change with the IPS represents a huge advance in accuracy. For further proof of this, we conducted a direct comparison.

## 3. Experimental Methods and Results

We fabricated a 3.5 MHz inductive long-stroke position sensor, whose main component was ZMID5201 with simulation and post calibration [36], which can achieve the compensation of offset, gain and linearization with the simulation tool. Subsequently, we conducted a comparative performance test of our sensor with a Hall-based 3D sensor, whose main IC components were A1335 (a 3D CVH sensor) and MLX90333 (a 3D Hall sensor).

We compared the accuracy of the sensors with changes of AG in the LS condition, and their immunity to external magnetic disturbances (15 Hz and 1 kA/m, following the ISO11452-8 standard). In addition, we conducted a comparative test under 0.1 T particle-contaminated conditions, which could be imposed in the *x*, *y*, or *z* directions of the sensor with a Helmholtz coil.

### 3.1. Test Setup for Gradiometric Arctangent 3D Hall Sensor

We set identical targets moving lengthwise over a range of 12 mm to compare the effect of AG and external fields on the accuracy of 3D Hall sensors and IPSs. We used identical airgaps.

**Figure 7:**
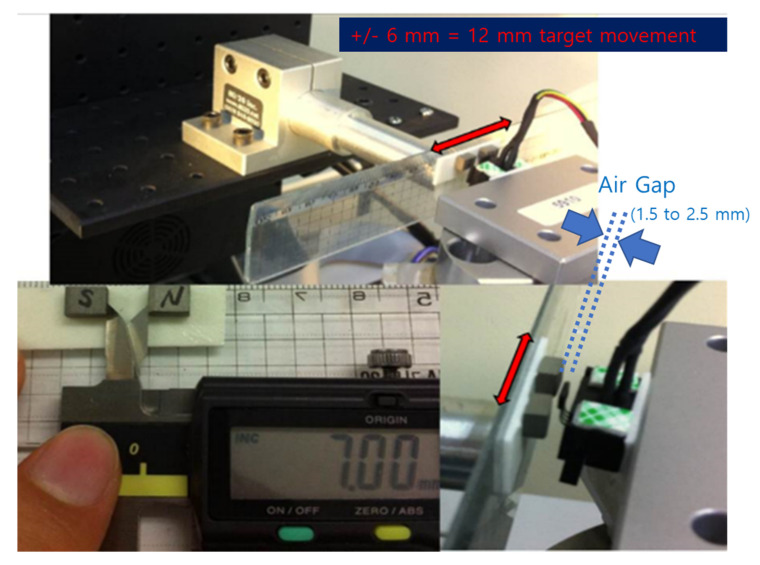
Test setup for 3D Hall sensors with 12 mm range of magnet position.

The accuracy specifications, moving range, and airgaps were set to match actual automotive long-stroke position sensor conditions. An automotive 12 mm position sensor should be accurate within ±1% for the full-scale target moving range. The system was operated at 25 °C. The magnet or target moving range was ±6 mm (or 12 mm). The airgaps were 1.5, 2.0, and 2.5 mm (Figure 7). The Hall-based LS gradiometric 3D arctangent sensors had 0.18 T samarium–cobalt magnets with the dimensions 6.5 × 4 × 4 mm (Figure 7).

### 3.2. Test Results for Gradiometric Arctangent 3D Hall Sensor

**Figure 8:**
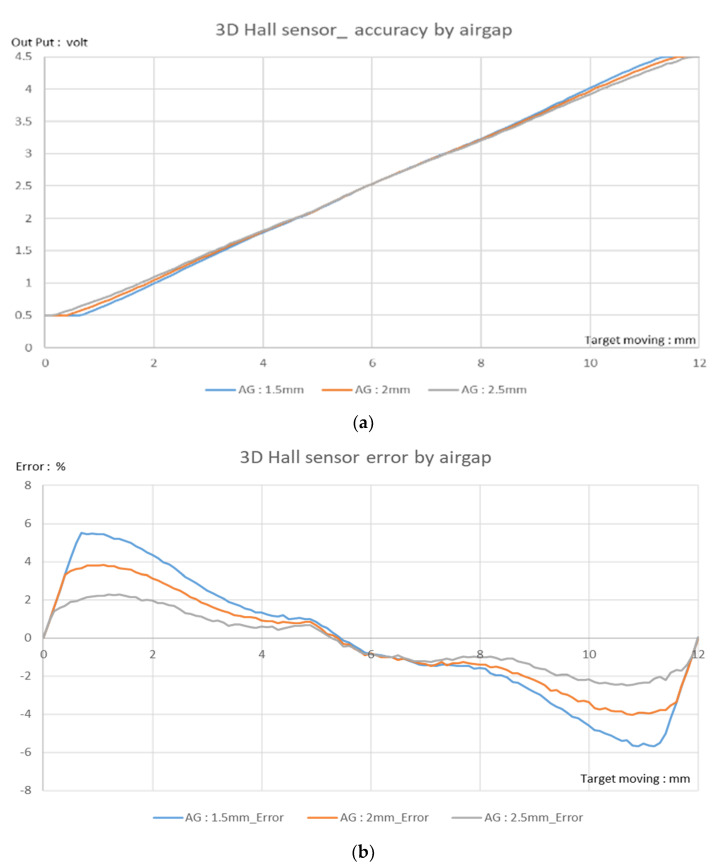
Results of 3D Hall sensor (MLX90333) 0–12 mm-stroke test for various airgaps (AG): (**a**) output vs. target position; (**b**) corresponding error. The accuracy requirement of ±1% was not met.

The 3D Hall sensor test results, calibrated for a 12 mm moving range, are shown in Figure 8. A 3D sensor with the same distance between magnets (7 mm) and the same moving range (*x*-axis, ±6 mm, or 12 mm) could not meet a test-accuracy performance specification of ±1% even using the arctangent function; the actual result was over ±5% FSO. This accuracy was consistent with expectation: the raw data at a long distance decreased dramatically down to the noise level.

### 3.3. Test Setup for Arctangent IPS

**Figure 9:**
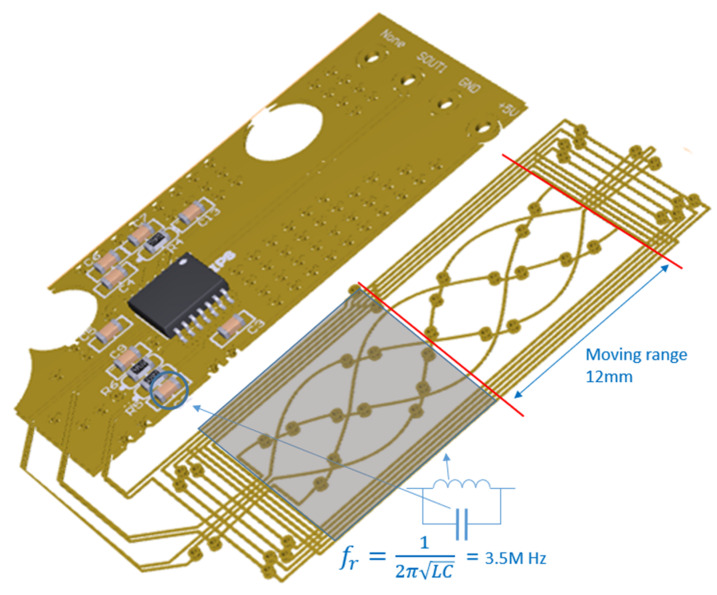
Inductive long-stroke position sensor with metal target size (blue square) and actual target moving range 12 mm.

In the case of the IPS, half the PCB pattern comprised the moving range. Therefore, the moving range was 12 mm, as shown in Figure 9.

The test conditions for the Hall-based and inductive-based sensors were set to ensure that the same target range conditions (i.e., 12 mm and ±6 mm) were implemented, as shown in Figure 9.

We fabricated an *x*, *y*, and *z* motion controller with a resolution of 0.0001 mm to determine the exact moving range. All moving ranges in vehicles (i.e., 5–100 mm) could be realized using this machine; in the experiment, the target was moved from 0 to 12 mm, the same distance as the moving range in Hall sensor applications.

**Figure 10:**
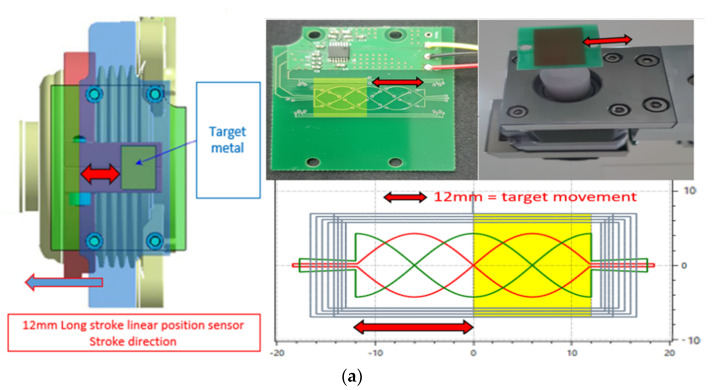

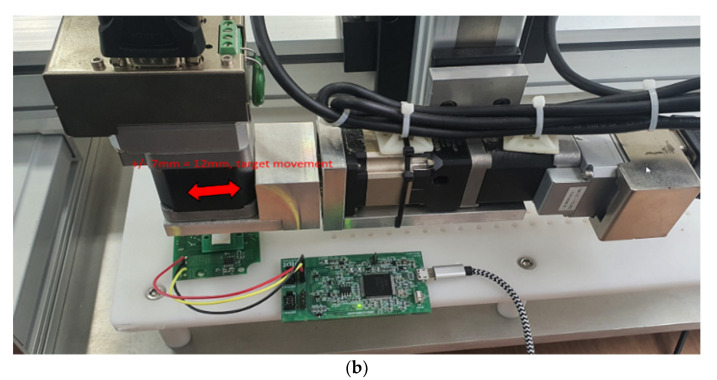
Test setup for long-stroke inductive position sensor (IPS). (**a**) PCB shape with vehicle sensor and target (yellow) shape. (**b**) Actual test equipment and IPS PCB layout.

The IPS was tested with the same airgap as the 3D Hall sensor. The test setup is shown in Figure 10. The 12 mm moving range is denoted by the red arrow in the Figure 10.

### 3.4. Test Results for IPS

**Figure 11:**
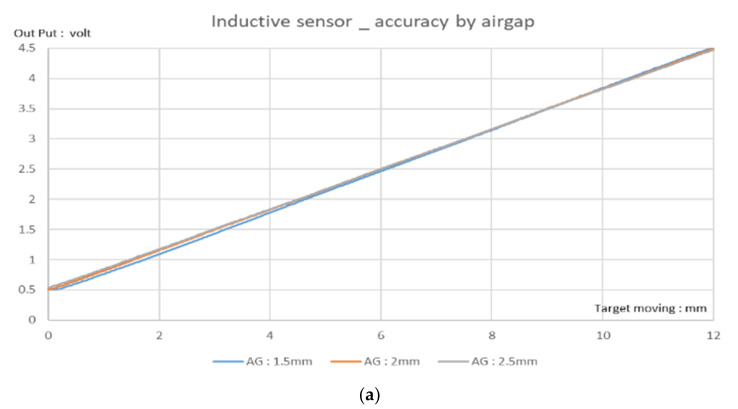

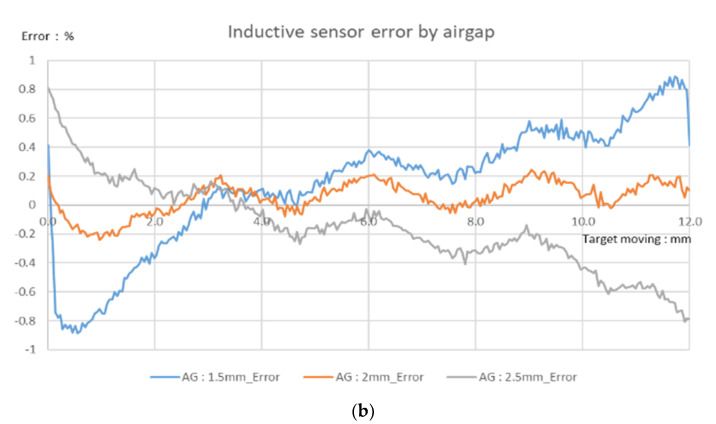
Stroke test results for ZMID5201 inductive position sensor (IPS): (**a**) Output voltage vs. distance for different airgaps (AGs). (**b**) Corresponding error result. IPS meets the error limit of ±1%; the actual error is within ±0.85% at room temperature (25 °C).

The accuracy of the IPS was within ±0.85% at the full-scale range (0–12 mm), including the airgap change (1.5–2.5 mm), as shown in Figure 11a,b. This error is much less than that of the Hall-based 3D sensor. These radiometric $Pv_{a}$ measurements between come from almost unchanged raw data of arctangent operation, rather than the changed data used by the 3D Hall-based sensor for distance and AG. The IPS application hardly affects the raw data, as shown in Figure 6b.

### 3.5. Test of Immunity to External Magnetic Fields

**Figure 12:**
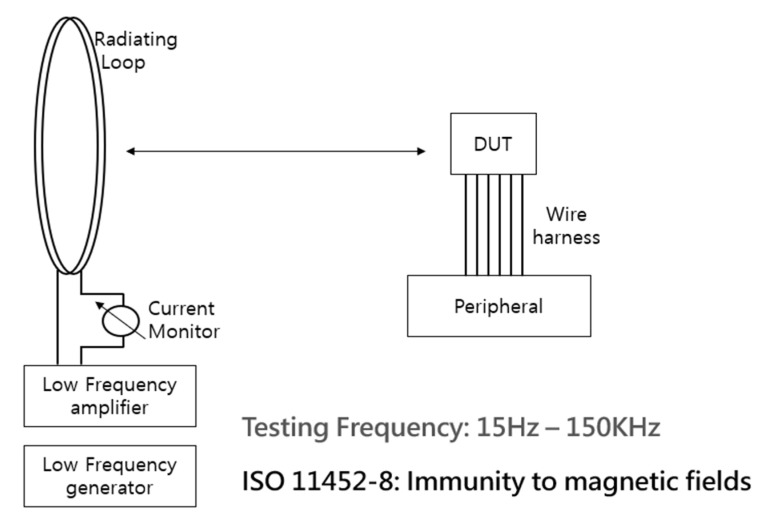
Schematic drawing of ISO11452-8 magnetic immunity test at 15 Hz and 1 kA/m. DUT: device under test.

**Figure 13:**
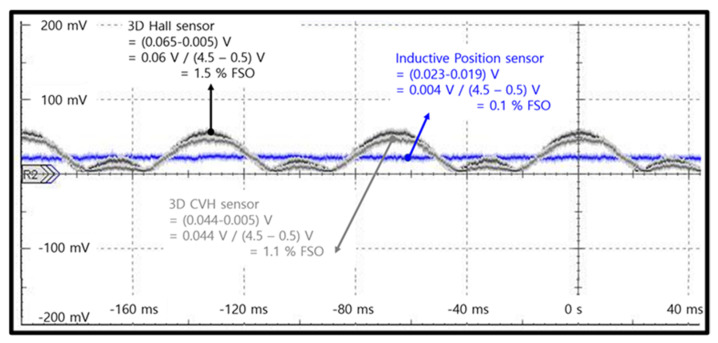
Comparison of immunity test results for sensors exposed to an external magnetic field: inductive position sensor (ZMID5201) and two 3D Hall sensors (MLX90333 and CVH (A1335)). Each was exposed to a 15 Hz and 1 kA/m magnetic field.

We performed standard ISO11452-8 magnetic immunity tests (Figure 12) to determine the impact of an EXMF. The specification for pass tolerance deviation was 1%. The acceptable full-scale voltage range was 0.5–4.5 V; the mean value should be less than 40 mV. Moreover, we tested the frequency profile of the external magnetic field in the proposed IPS and gradiometric arctangent 3D Hall sensors. The behavior of the IPS (denoted by the blue line in Figure 13) indicated high accuracy (within 1%). The full-scale value was within ±5 mV when exposed to a 15 Hz and 1 kA/m EXMF. By contrast, in 3D Hall sensors—the black and gray lines represent the MLX90333 (arctangent 3D Hall sensor) and A1335 (arctangent CVH Hall sensor), respectively—the deviation value was more than 60 mV. Therefore, the proposed IPS met the 1% external magnetic field immunity accuracy standard for a position sensor. For reference, 15 Hz at 1 kA/m corresponds to a magnetic field with a strength of 0.00126 T.

**Figure 14:**
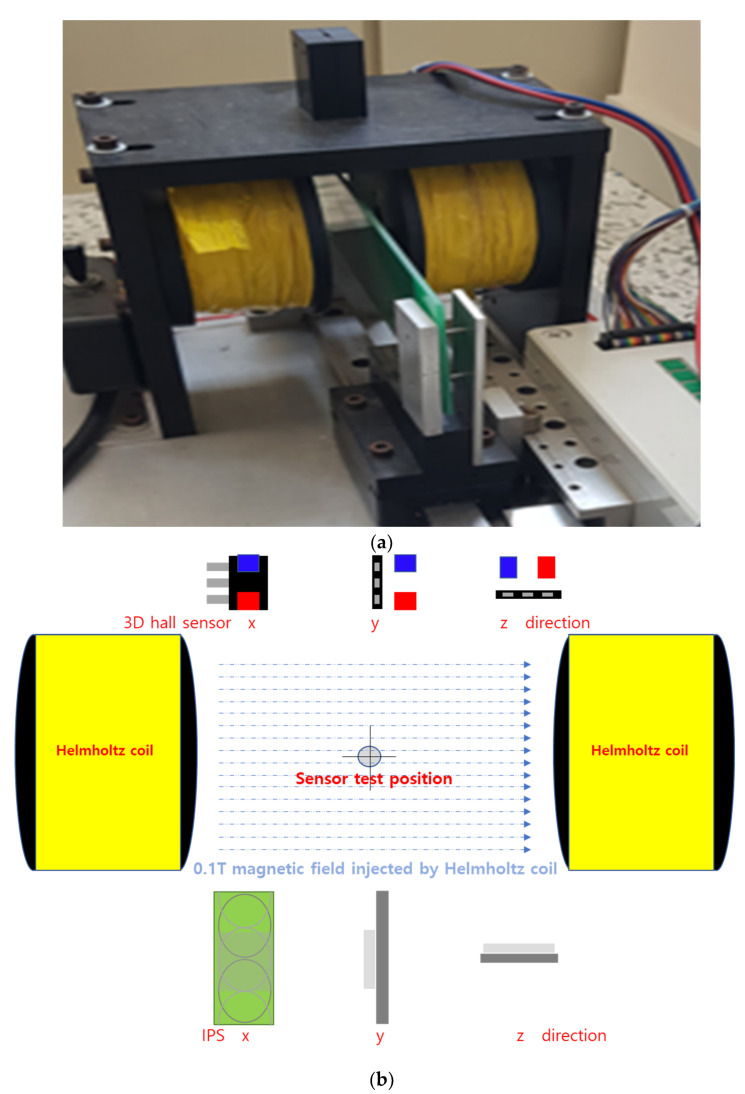
Experiment to determine the effect of a strong magnetic field (0.1 T) in *x*, *y*, or *z* directions on a 3D Hall sensor or inductive position sensor (IPS): (**a**) photograph of test setup; (**b**) schematic diagram.

For a more intuitive test, a stronger magnetic field can be used; such strong fields may sometimes occur in an actual vehicle through the effects of external particles. In this study, a Helmholtz coil was used to inject a 0.1 T magnetic field. First, each device was calibrated at the center target position, where *V*_out_ was 2.5 V. The device was then installed at the sensor test position (Figure 14). Through the Helmholtz coil, the 0.1 T external magnetic field was injected. Subsequently, the device’s outputs were checked (Table 1).

Regardless of whether the 0.1 T magnetic field was injected into the PCB pattern in the *x*, *y*, or *z* direction, the output of the IPS was always within ±1% (i.e., 0.075% to 0.28%). However, in the case of the 3D Hall sensor, the errors in different directions due to the inflow of the magnetic field and the device’s own position error were quite large. Note that in theory, there should be no B field at all in the *x* and *z* directions.

The difference in performance is because the IPS, operating at 3.5 MHZ, uses a completely different frequency domain from the 3D Hall sensor and is unaffected by magnetic fields with frequencies below 150 kHz. Therefore, the long-stroke IPS has more robust immunity against EXMFs than do 3D Hall-based sensors.

## 4. Conclusions

In terms of accuracy, the most significant limitation of existing gradiometric arctangent 3D Hall sensors is the large error caused by a change in the B field due to either a change in the LS position and AG or an external magnetic field under 150 kHz. Because the raw output of the 3D Hall sensor decreases exponentially in an EXMF, the error inevitably increases, even if the arctangent algorithm is adopted.

The arctangent 3D Hall sensor and 3.5 MHz arctangent IPS discussed in this study are compared in Table 2. The 12 mm moving range is mathematically converted to θ by the arctangent method, which was implemented using CORDIC computing and linearization calibration methods. For the IPS, this can achieve a ±0.85% accuracy error at a 12 mm LS moving range, with an AG of 1.5–2.5 mm. This result is significantly better than the 5.4% accuracy of the 3D Hall sensor.

From the point of view of immunity to EXMFs, our proposed long-stroke IPS position sensor is advantageous because it uses a high-frequency signal (3.5 MHz), effectively avoiding external magnetic fields in the low-frequency range (under 150 kHz) typical of the automotive environment. It can meet a recent ISO26262 safety standard, satisfying the goal of not missing the target position. By contrast, existing gradiometric arctangent 3D Hall sensors violate the ISO26262 safety goal.

The implemented sensor achieves the stable ±0.1% error performance under external magnetic fields (15 Hz and 1 kA/m) stipulated in the ISO11452-8 standard. In addition, in the 0.1 T high magnetic field test, the IPS demonstrated strong immunity to external magnetic fields.

Based on these advantages, the 3.5 MHz long-stroke IPS proposed in this study could be used in all automotive position-sensing applications that must meet the ISO26262 safety goal. It can be implemented in different types of vehicle position sensors, whether of rotary, arc, or linear type, particularly in LS applications with a stroke length >12 mm. It can also be used as a safety sensor that does not miss target positions and affords higher accuracy than existing sensors, including gradiometric 3D Hall effect sensors.

## Figures and Tables

**Table 1:** Errors caused by exposure to strong magnetic field.

Moving Range = 12 mm, Accuracy Spec. = within 1%/FSO FSO = (0.5–4.5 V)	Calibrated on Center Position	Exposed to 0.1 T by Helmholtz Coil		
		*x* Direction	*y* Direction	*z* Direction
Arctangent CORDIC 3D Hall sensor	2.5 V 0% error	4.0 V 37.5% error	4.5 V 100% error Target loss	4.2 V 42.5% error
3.5 MHz Arctangent CORDIC IPS	2.5 V 0% error	2.497 V 0.075% error	2.489 V 0.28% error	2.491 V 0.23% error

**Table 2:** Comparison of 3D Hall sensor to proposed IPS.

Moving Range = 12 mm Accuracy Spec. = Within 1%/FSO (Full-Scale Output)	Accuracy Error Under Airgap Change 1.5/2/2.5 mm	Exposed to External Magnetic Field 0.00126 T, 15 kHz	Exposed to 0.1 T by Helmholtz Coil
Arctangent 3D Hall sensor	Max. 5.4% error Figure 8	Max. 1.5% error Figure 13	Max. 100% error (position loss) Table 1
Main error source	B-field exponential wave form in airgap and additional 12 mm stroke movement	Theoretically affected by all external magnetic fields under 150 kHz.	
3.5 MHz Arctangent IPS	Max. 0.85% error Figure 11	Max. 0.1% error Figure 13	Max. 0.28% error Table 1
Main error source	B-field change by airgap	Uses 3.5 MHz instead of automotive environment frequencies (which are under 150 kHz); not affected by external magnetic field.	
	For IPS, error effect of airgap is greater than that of external magnetic field.		
**Over-12 mm Long-Stroke Application**	**Strong Point**	**Weak Point**	
Arctangent 3D Hall sensor	<12 mm application, size small and can meet an accuracy 1%	Low accuracy; uses strong magnet (high cost); low EXMF immunity. Cannot meet safety goal; loses target position in strong magnetic field.	
3.5 MHz Arctangent IPS	Safety; high accuracy; strong EXMF immunity. Can meet safety goal; does not lose target position in strong magnetic field.	Needs larger PCB pattern size than 3D Hall sensor.	

## Data Availability

Not applicable.
